# The Enigmatic World of Fungal Melanin: A Comprehensive Review

**DOI:** 10.3390/jof9090891

**Published:** 2023-08-31

**Authors:** Malika Suthar, Laurent Dufossé, Sanjay K. Singh

**Affiliations:** 1 National Fungal Culture Collection of India, Biodiversity and Palaeobiology Group, MACS-Agharkar Research Institute, G.G. Agarkar Road, Pune 411004, India; malikasuthar@aripune.org; 2 Faculty of Science, Savitribai Phule Pune University, Ganeshkhind Road, Pune 411007, India; 3 Laboratoire de Chimie et Biotechnologie des Produits Naturels (ChemBioPro), ESIROI Agroalimentaire, Université de La Réunion, F-97400 Saint-Denis, France

**Keywords:** melanin, bioactive pigment, biotechnological potential, fungi

## Abstract

Synthetic dyes are generally not safe for human health or the environment, leading to the continuous search and growing demand for natural pigments that are considered safer, biodegrade more easily, and are environmentally beneficial. Among micro-organisms, fungi represent an emerging source of pigments due to their many benefits; therefore, they are readily viable on an industrial scale. Among all the bioactive pigments produced by fungi, melanin is an enigmatic, multifunctional pigment that has been studied for more than 150 years. This dark pigment, which is produced via the oxidative polymerization of phenolic compounds, has been investigated for its potential to protect life from all kingdoms, including fungi, from biotic and abiotic stresses. Over time, the research on fungal melanin has attracted a significant amount of scientific interest due to melanin’s distinct biological activities and multifarious functionality, which is well-documented in the literature and could possibly be utilized. This review surveys the literature and summarizes the current discourse, presenting an up-to-date account of the research performed on fungal melanin that encompasses its types, the factors influencing its bioactivity, the optimization of fermentation conditions to enhance its sustainable production, its biosynthetic pathways, and its extraction, as well as biochemical characterization techniques and the potential uses of melanin in a wide range of applications in various industries. A massive scope of work remains to circumvent the obstacles to obtaining melanin from fungi and exploring its future prospects in a diverse range of applications.

## 1. Introduction

The negative consequences that synthetic dyes have on the environment and human health are well known. Therefore, the toxic effects caused by these synthetic chemicals and their industrial byproducts on human health and the environment represent a developing concern [1,2,3]. As a result, a critical demand for the extraction of pigments from natural sources, mostly micro-organisms, is expanding globally. Among micro-organisms, fungi comprise a group that is significant for the production of pigments because when grown on a large scale, they produce high yields of metabolites, serving as “microbial cell factories” and making the bioprocess commercially viable [4]. Amid the wide range of pigments produced by fungi there is a significant class of pigments called melanins, the production of which has been known about for more than 150 years [5,6,7,8]. A melanin is a secondary metabolite produced by fungi which contributes to the survival mechanism of fungi in unfavorable environments [9].

The term “melanin” is derived from the Greek word “melanos”, meaning “black”, and it was first used by the Swedish chemist Berzelius in 1840 [10,11]. Melanins are usually dark-brown- or black-colored pigments of high molecular weights which are negatively charged, hydrophobic in nature, and formed via the oxidative polymerization of phenolic or indolic compounds [12]. These pigments are widely synthesized by plants, animals, fungi, protists, protozoans, pathogenic bacteria, and helminths [13]. However, melanin is known for its distinctively unique and exceptional physicochemical and biological properties, such as its black color, insolubility in organic solvents, resistance to acid degradation, reduction of silver nitrate ammonia solutions, and provision of positive reactions to polyphenols [14]. It is the stability, insolubility, and resistant nature of melanin that make fungi capable of surviving in unfavorable environments, but these qualities also make it difficult to work with, limiting the analytical approaches that successfully identify and characterize the pigment.

Fungal melanin is generally located either extracellularly (secreted outside, in the environment) or intercellularly (in the cell wall). However, according to Agustinho and Nosanchuk [15], the distribution of melanin in the cell structure varies between species.

While melanin is sometimes found to be associated with the matrix, fibrillar in nature, and extending out of the cell wall, in other fungi, it is generated as a discrete and strongly delimited layer [16].

This pigment, on the other hand, demonstrates a variety of biological and functional actions, including defending the fungi from oxidative damage, UV radiation, enzymatic lysis, and extremely hot and cold temperatures. It serves as a physiological redox buffer, providing structural rigidity to cell walls and protection against antimicrobial drugs [17]. Melanin has been referred to as “fungal armour”, conferring advantages to fungi so they may survive in harsh conditions and increasing the virulence of a variety of fungi that are pathogenic to plants and humans [18].

In recent years, the multifarious biological and functional properties of melanin have been well documented in the literature and have attracted keen scientific interest. Therefore, a comprehensive analysis is required to understand the significance and current state of fungal melanin research on a global scale. In this review, we attempt to understand the various types of melanin, the factors influencing its bioactivity, the optimization of fermentation conditions to enhance its sustainable production, its biosynthetic pathways, its extraction and biochemical characterization techniques, and the potential uses of melanin in a wide range of applications in the bioelectronics, pharmaceutical, dermocosmetic, food processing/packaging, and dyeing industries. This study also attempts to shed light on the challenges currently facing research on fungal melanin and provides recommendations for possible future perspectives.

## 2. Types of Melanin

With specificity to the fungal kingdom, types of melanin are classified as eumelanins, pheomelanins, allomelanins (DHN-melanins), and pyomelanins. These classifications are based on the chemical composition of the monomer subunit structure of the pigment and are widely accepted [19]. Both eumelanins and pheomelanins are derived from the common precursor dopaquinone, which is obtained via the oxidation of tyrosine or L-Dopa.

**Eumelanins** are dark brown or black pigments containing 6–9% nitrogen and 0–1% sulfur. Their precursor, dopaquinone, undergoes cyclization, resulting in cyclodopa, which is rapidly oxidized into dopachrome. Dopachrome is then converted into units of 5,6-dihydroxyindole (DHI) and 5,6-dihydroxyindole-2-carboxylic acid (DHICA) to form eumelanins.

Some examples include *Auricularia auricula*, *Cryptococcus neoformans*, *Candida albicans*, *Paracoccidioides brasiliensis*, and *Sporotrix schenckii* [20].

Pheomelanins are yellow-red pigments containing 8–11% nitrogen and 9–12% sulfur and are composed of benzothiazine monomer units. In the presence of cysteines, dopaquinones connect with cysteines to form 5-s cysteinyl dopa and 2-s cysteinyl dopa, which provide benzothiazin and benzothiazole intermediates that polymerize to produce pheomelanins.

Some examples include *Auricularia auricula* and *Tuber melanosporum* [20].

Allomelanins are a heterogenous group of nitrogen-free polymers produced through the polymerization of di-DHN (1,8-dihydroxynaphthalene). Polyketide synthase (PKS) catalyzes the initial step of the pathway in which it converts the precursor malonyl CoA into 1,3,6,8-tetrahydroxynaphthalene (THN).

Some examples include *Auricularia auricula*, *Aspergillus fumigatus*, *Phoma* sp., *Alternaria alternata*, *Cladosporium carionii*, and *Fonseceae pedrosoi* [21].

Pyomelanins involve tyrosine transaminase forming 4-hydroxyphenylpyruvate, which is converted into homogentisic acid by dioxygenase. The dioxygenase is spontaneously oxidized into benzoquinone acetate and polymerized to form a pyomelanin.

Some examples include *Penicillium chrysogenum*, *Sporothrix schenckii*, *Aspergillus fumigatus*, *A. kawachii*, *Madurella mycetomatis*, and *Yarrowia lipolytica* [21].

Glutaminyl-4-hydroxybenzene melanin (GHB) is another type of melanin that has been observed in the basidiospore wall of *Agaricus bisporus*, in which “browning” has been reported to be a common phenomenon wherein the melanogenic phenols are enzymatically converted into quinones and then evolve into melanins [22].

The precursor GHB (glutaminyl-4-hydroxybenzene) is converted into the immediate precursor of the spore-wall melanin, GDHB (glutaminyl-3,4-dihydroxybenzene). GDHB is found only in reproductive hyphae, which eventually form melanized spores [23].

However, many basidiomycetous mushrooms have been reported to contain GHB, which concludes that while DHN melanin might be specifically produced by ascomycetous fungi, GDHB melanin is only produced by basidiomycetous fungi [23].

## 3. Biosynthesis

**Figure 1:**
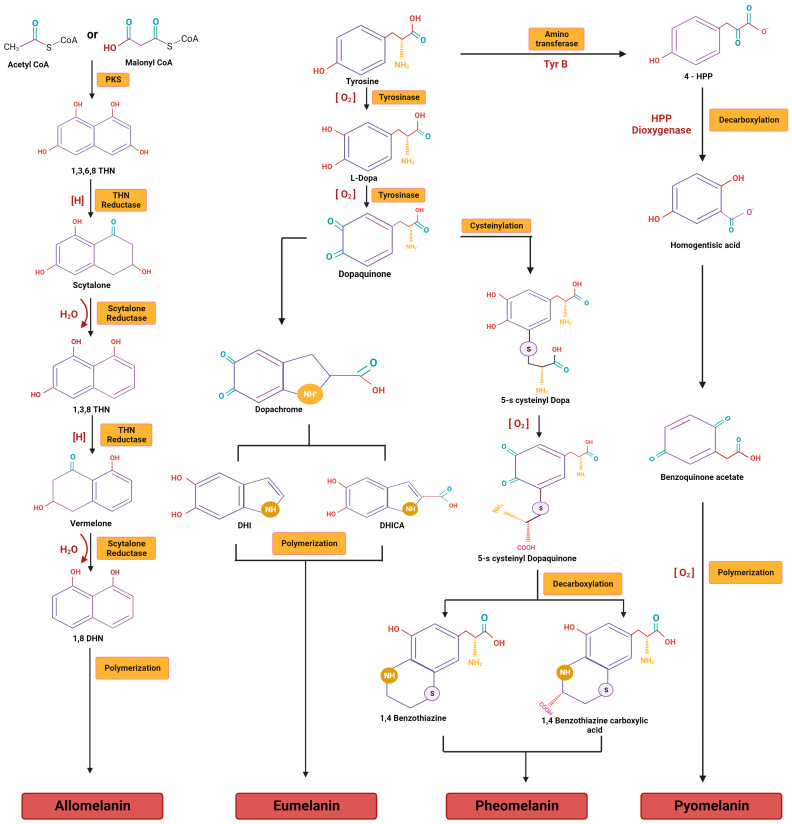
Biosynthetic pathways for various types of fungal melanins.

Specific to fungal melanin, based on precursors and the pathway taken, the two most significant types of melanin are DHN-melanin (1,8-dihydroxy naphthalene) and DOPA-melanin (L-3,4-dihydroxyphenylalanine) (Figure 1). Pathogenesis has been linked to both forms of melanin [24].

### 3.1. DHN Melanins

Most ascomycetes and deuteromycetes synthesize the 1,8-dihydroxy naphthalene (DHN) type of melanins through the polyketide pathway, using 1,8-DHN as a precursor. A general model for the biosynthesis of DHN-melanins has been explained below; however, it varies in different fungi [25].

As the initial step in the process of biosynthesis, malonyl-CoA/Acetyl CoA is converted into 1,3,6,8-tetrahydroxynaphthalene (1,3,6,8-THN) by the enzyme polyketide synthase (PKS). The enzymatic dehydration of scytalone results in 1,3,8-trihydroxy naphthalene (1,3,8-THN), which is then further reduced to vermelone by a second reductase enzyme. The scytalone dehydratase catalyzes a further dehydration phase that results in the intermediate 1,8-dihydroxy naphthalene (DHN). A laccase subsequently catalyzes the dimerization and finally polymerizes the molecules to create DHN-melanins [26].

Inhibitor action: tricyclazole inhibits the two-step dehydrogenation reaction that occurs during the formation of DHN-melanin, which involves reducing THN to scytalone and 1,3,6-trihydroxynaphthalene to vermelone. Another enzyme involved in the DHN pathway, tetrahydroxynaphthalene reductase, is inhibited by pyroquilon, which prevents the production of melanin [27]. Additionally, 1,3,6-trihydroxynaphthalene reductase is also inhibited by carpropamid and fenoxanil as a single dehydrase in the melanin production pathway [28].

### 3.2. DOPA Melanins

Conversely, basidiomycetes and other imperfect fungi produce DOPA melanins, typically using L-DOPA as a precursor, similar to the mammalian pathway. The melanins formed from DOPA are eumelanins and pheomelanins [29].

With respect to the biosynthesis of DOPA melanins, phenol oxidases form a second major group of enzymes that produce pigments, and they fall into two subgroups—laccases and catechol oxidases (commonly called tyrosinases)—on the basis of the differences in their substrate specificities. However, both have copper ligands and require bound copper ions for their activity [30]. In general, laccases catalyze the one-step oxidation of dihydroxyphenols into quinones, while tyrosinases catalyze the two-step oxidation of tyrosine [31].

The synthesis of DOPA-melanin in fungi has been of scientific interest for a long time. Numerous fungi such as *Neurospora crassa*, *Podospora anserina*, *Aspergillus nidulans*, and *Aspergillus oryzae*, as well as pathogenic fungi like *C. neoformans*, have been studied in this regard [32].

Depending on the precursor molecule, either L-dopa or tyrosine is converted to produce dopaquinone. The process is catalyzed by tyrosinase, which acts as the primary rate-limiting enzyme of the reaction. Then, dopaquinone undergoes a series of reactions to form a eumelanin or pheomelanin, respectively [33].

Dopaquinone is a highly reactive intermediate. In the absence of thiols, dopaquinone forms leucodopachrome, which is then oxidized into dopachrome. Hydroxylation (and decarboxylation) yields dihydroxy-indoles, which can polymerize to form DOPA-melanins [34].

Inhibitor action: the activity of the rate-limiting enzyme tyrosinase can be inhibited, preventing the production of DOPA-melanin by using kojic acid, which chelates Cu^2+^ at the active site of tyrosinase and prevents the formation of 5,6-dihydroxyindole-2-carboxylic acid (DHICA) from dopachrome [35]. Similarly, azelaic acid also inhibits the action of tyrosinase at the binding site, effectively decreasing the formation of DOPA-melanin by binding to amino and carboxyl groups [36].

## 4. Optimization for Enhancing Pigment Production

The selection of appropriate fungal strains and the optimal culture conditions (suitable media, temperature, pH, and aeration) for pigment production are essential for effective fermentation. In this regard, filamentous fungi are favored sources of pigments as they may be grown under a wide range of cultivation conditions. They can grow in solid and submerged culture systems, and they have the potential to be used in large-scale industrial fermentations to produce a rational yield of pigment [37].

Nutritional and physical factors also have significant impacts on pigment production. According to studies, complex media such as potato dextrose (PD) and malt extract (ME) favor increased pigment production because they involve nutrients that regulate gene expression and activate metabolic pathways [38].

*Microdochium bolleyi*, first known as *Gleosporium bolleyi* and later as *Aureobasidium bolleyi*, is a common soil fungus, and it was observed that media composition and light are the two elements that influence the formation of the pigment melanin in this fungus. Pigment production was found to be at a maximum under alternating light and dark rhythms in the case of light, while among all the media investigated, PDA was able to significantly change its colony differentiation (i.e., from hyaline cells to melanin-pigmented chlamydospores) and create intense pigmentation [39].

Since pigments are secondary metabolites that are generally produced during a late growth phase (idiophase), an increase in mycelial growth does not necessarily imply an increase in pigment production. For example, when the carbon source best suited to melanin production in *Penicillium* sp. was investigated, it was found that fructose produced the maximum mycelial growth, while the maximum pigment production was achieved with starch [40].

Similarly, temperature and pH have significant impacts on the fungal production of melanin. The maximum pigment production in *Monascus purpureus* was observed at 30 °C, whereas the yield was substantially lower at 37 °C [41]. In wood-inhabiting fungi, like *Trametes* and *Xylaria*, the maximum melanin pigmentation was produced at a pH range of 4.5–5. This happens because the effects of pH and temperature change the protein activities of the fungal cell [42].

The pigment output generated during fermentation is also associated with proper aeration relating to O_2_ dependency. For example, the maximum pigment production was achieved in *M. ruber* at an aeration rate of 0.05 L/min; thus, this aeration rate was found to be suitable for oxygenating the fungus [43]. This suggests that the formation of this pigment requires the oxidative polymerization of the precursors.

Apart from the nutritional and physical factors involved in fermentation, the use of low-cost media has always been believed to be a convenient and cost-effective method for the production of large amounts of melanin. Agro-industrial residues have been studied and found to be successfully utilized as substitutes for expensive substrates that support fungal growth and pigmentation [44]. For example, the use of corn cob powder as a substrate for the production of pigments by *M. purpureus* resulted in an increase in pigment production [45]. Experiments conducted for the optimization of the media used in submerged cultures of *Auricularia auricula* for the large-scale production of melanin demonstrated that glucose, tyrosine, peptone, and CaCO_3_ had significant effects on the biosynthesis of melanin, and a 3.29-fold increase in melanin production could be observed [46]. Pyrocatechol, tyrosine, and copper ions were also found to enhance melanin production in submerged fermentation conditions in *Inonotus obliquus* [47].

According to a study by Jalmi et al. [48], the supplementation of tyrosine to *Gliocephalotrichum simplex* produced a significant amount of extracellular melanin (6.6 g/L), which may also have potential applications in biotechnology. Similarly, when the optimum conditions for an increased yield of melanin produced in *Spissiomyces endophytica* were investigated, it was found that the highest fungal pigment yield was observed in a GYEP medium at pH value of 6 and at 25 °C over 3 weeks of cultivation [49].

When the optimum conditions for the ultrasonically assisted extraction of melanin from *Sporisorium reiliana* were investigated via RSM, it was found that a combination of power (245 W), temperature (50 °C), time (2 h), and NaOH extract (pH 13) increased the production of melanin in this fungus by 3.52% [50].

## 5. The Functional Role of Fungal Melanin

### 5.1. The Role of Melanin in Anti-Microbial Effects

The role of melanin as an anti-microbial agent is also interesting as it exhibits significant anti-bacterial and anti-fungal activities. For example, Arun et al. [51] reported that the mushroom fungus *Schizophyllum commune* produces melanin with an effective anti-bacterial activity against *E. coli*, *Proteus* sp., *Klebsiella pneumonia*, and *Pseudomonas fluorescens* and anti-fungal activity against *Trichophyton simii* and *T. rubrum*. Silver nanoparticles imbued with melanin from *Yarrowia lipolytica* exhibited an antimicrobial activity against the pathogen *Salmonella paratyphi* [52].

Similarly, the production of melanin from the endophytic fungus *Phoma* sp. RDSE17 was also studied to exhibit anti-bacterial potential against *Bacillus subtilis*, *Staphylococcus aureus*, *E. coli*, and *Salmonella typhi* and anti-fungal potential against *A. flavus*, *A. niger*, *Rosellinia* sp., and *Ustilaginoidea virens* [53]. *Auricularia auricula* melanin also has a high rate of inhibiting biofilm formation, acting against *E. coli* K-12, *Pseudomonas aeruginosa* PAO1, and *P. fluorescens* P-3 [54]. Melanin from *Lachnum YM30* also exhibits an effective inhibitory effect on Gram-positive bacteria like *Listeria monocytogenes* and *Staphylococcus aureus*, as well as Gram-negative bacteria like *Escherichia coli* and *Vibrio parahaemolyticus* [55].

According to Lopusiewicz et al. [56], pure melanin extracted from *Armillaria mellea* also exhibited antibacterial activity against *Bacillus cereus*, *B. subtilis*, *E. faecalis*, and *P. aeruginosa*. Likewise, it has also been noted that the melanin of *Exidia nigricans* and *Scleroderma citrinum* possesses antibacterial actions against *E. faecalis* and *P. aeruginosa* [57,58].

### 5.2. The Role of Melanin in Photoprotection

Prolonged exposure to the sun may lead to skin burns and the suppression of the immune system. The synthetic chemicals used in sunscreen products, such as benzophenone-3, cinnamate, and octocrylene, produce free radicals that cause damage to the skin [59]. Among all the biological pigments known, melanin has the ability to absorb all visible wavelengths. Hence, these pigments are found to act as UV light shields, and it has been confirmed via human experience that human DOPA-melanin protects the skin from sunlight. This suggests that melanin has anti-radiation properties, a significant application of the pigment [60].

*Cladosporium* sp., *Alternaria alternata*, *Aureobasidium pullulans*, and *Hormoconis resinae* are some of the melanized fungi that have been observed to colonize the damaged Chernobyl nuclear reactor, which has a strong radiation field [61].

The microscopic melanized fungi *Cryomyces antarcticus* and *Cryomyces minteri* also exhibit high levels of UV resistance for a few hours compared to their non-melanized strains [62].

*Lachnum*, a saprophytic fungus, has been found to have excellent anti-radiation activity and to resist UV radiation against the bacteria *E. coli* and *S. aureus* as well as the yeast *Saccharomyces cerevisiae* [63]. Additionally, it has been suggested that it slows the aging process by preventing the production of lipid peroxidation products during the oxidative metabolic process. As a result, it may be used as a natural anti-radiation ingredient in sunscreens and as an anti-aging medication in dermocosmetic industries [64].

### 5.3. The Role of Melanin in Thermoregulation

Studies suggest that the melanin-producing ability of fungi has been closely linked to their ability to survive under extreme conditions. These darkly pigmented polymers protect the cells against various stresses imposed by the environment and play an important role in thermoregulation, e.g., in *C. neoformans*, melanin protects the fungi against temperature extremes, making them thermostable; therefore, the fungi are able to survive in extreme heat (42–47 °C) and in cold (−20 °C) temperatures. This activity was studied by exposing both melanized and non-melanized *C. neoformans* cells to extreme heat and cold; it was then concluded that melanized cells were less susceptible to heat and cold compared to the non-melanized cells [65].

According to studies, melanin confers protection against extremes of hot and cold on *Exophiala (Wangiella) dermatitidis* [66]. The presence of melanin in the hyphae of *Phyllosticta capitalensis*—a cosmopolitan foliar endophyte—is also considered to be responsible for the survival of this fungus even under stressful conditions [67].

### 5.4. The Role of Melanin in Protection from Radiation

Due to its chemical structure, the presence of stable free radicals, and its spatial arrangement, melanin has also been observed to protect against extremely high levels of ionizing radiation. One of the classic examples of fungal melanins surviving in an extreme environment are the microbiota at the site of the Chernobyl nuclear reactor accident. More than 37 different kinds of fungi were found within the damaged reactor, and a significant number of black fungi were discovered in the polluted soil near the Chernobyl Nuclear Power Plant. Even though the radiation levels were increased manifold and were lethal in nature, fungi like *Alternaria alternata* and *Cladosporium sphaerospermum* were found on site. According to studies, this accident demonstrates that due to melanogenesis, fungi can survive in extreme (radiation-tolerant) environments. Upon conducting a genetic analysis, it was also observed that different fungal strains within the reactor revealed significant homology, while those outside the contaminated region were genetically diverse [68].

### 5.5. The Role of Melanin in Protecting against Oxidative Damage

Melanin enhances the survival of fungi by neutralizing oxidants caused due to stress in the surrounding environment. There have been many reports suggesting that the melanins produced by ascomycetous fungi such as *C. neoformans*, *Aspergillus nidulans*, *Aspergillus bridgeri*, and *A. alternata*, as well as basidiomycetes like *Exophiala pisciphila*, *Wangiella dermatitidis*, *Schizophyllum commune*, and *Inonotus hispidus*, exhibit potent antioxidant activity [69].

Łopusiewicz et al. [56,57,58] also isolated and characterized melanin from *Armillaria mellea*, *Exidia nigricans*, and *Scleroderma citrinum* in raw and purified forms, demonstrating its antioxidant properties. It was observed that the purified form of melanin had greater antioxidant activity than its raw form.

Fungal melanin is a powerful antioxidant due to its unique electrochemical properties, which allow it to act as an electron donor and electron acceptor [70]. The antioxidant properties of fungal melanin are attributed to the addition of free radicals, probably because melanin has unpaired electrons that cause it to interact with peroxyl radicals [71].

*Hypoxylon archeri* melanin has a better ability to protect 80.95% 5-thio-2-nitrobenzoic acid (TNB) from oxidative damage by H_2_O_2_ than synthetic melanin. It also has a better effect when scavenging hydrogen peroxide oxygen radicals and promotes the production of other fungal polyphenol oxidases [72].

Similarly, melanin from *Ophiocordyceps sinensis* has also proven to be an effective DPPH scavenger compared to BHT (butylated hydroxyl toluene) and alpha-tocopherol, which are commercial antioxidants. It has also displayed a much better ferrous-ion-chelating ability than a water extract, making it a strong ferrous ion chelator [73].

The melanin extracted from *Lachnum singerianum* has inhibited lipid peroxidation and slowed down the aging process of this basidiomycete, suggesting that this pigment could be used as a potential anti-aging drug [74].

### 5.6. The Role of Melanin in Metal Chelation

There are various functional groups present in the structure of melanin which provide an array of multiple nonequivalent binding sites for metal ions. Due to this property, the pigment is able to bind to heavy metals that are potentially toxic to cells. In addition, if useful metals that are important to the cell physiology of a fungus but are present in fewer amounts in the environment, melanin can concentrate them in a manner that makes them available to the cell in sufficient amounts, thus conferring an important advantage to the survival of melanized fungi [75].

For example, melanin pigments from the rhizomorphs of four *Armillaria* species (*A. ostoyae*, *A. calvescens*, *A. gemina*, and *A. sinapina*) were studied and demonstrated to adsorb Al, Zn, Fe, Cu, and Pb cations such that these ions were found to be 50–100 times more focused in the rhizomorphs than in the soil [76].

Similarly, the numbers of the melanin-producing fungi *Cladosporium* and *Alternaria* were also observed to gradually increase at industrial and roadside locations as they are resistant to contamination with heavy metals [77], e.g., the melanin in *C. neoformans* makes these fungi less susceptible to silver nitrate, which is reportedly highly toxic to fungi and bacteria; hence, they also protect the environment from heavy metal toxicity [78]. Similarly, melanin from *Ophiocordyceps sinensis* has also been studied to be an effective iron chelator [79].

Melanin also possesses the ability to chelate metal, which enhances its biosorption capacity, and they remove rare earth elements from wastewater via metal complexing. Hence, in this manner, they provide an alternative means of cleaning industrial effluents from wastewater [80].

## 6. The Extraction and Purification of Melanin

It is quite taxing to study this “enigmatic” pigment despite the promising and diverse attributes that it is known for. Melanin is highly ubiquitous and multi-functional in nature, but it is challenging to study and analyze this pigment due to its amorphous and insoluble nature.

One of the main factors determining the selection of a method of extracting melanin from a fungal cell is its localization in the cell. Melanin may be located extracellularly or inside the cell wall. The extraction of extracellular melanin generally involves acid precipitation. In the case of intracellular melanin, the cellular matrix is initially treated with an alkali (1 M of NaOH, 1 M of KOH, or 0.5 M of NH_4_OH), which is then followed by autoclavation. Next, the acid precipitation step occurs in which melanin is extracted by boiling the cellular matrix in acid for several hours [81].

Even after the successful extraction of crude melanin, other biological components such as proteins, carbohydrates, and lipids occur in close affinity/binding. Therefore, the purification of crude melanin is important, which involves successively washing the crude fungal melanin with water and other different organic solvents (chloroform, ethyl acetate, and ethanol) [82].

However, studies revealed that this conventional alkali–acid extraction method employed for melanin involves harsh treatments, and the melanin is significantly disrupted during the process. The method is also time-consuming and produces low melanin yields [83].

Hence, with concerted efforts over time, advanced extraction methods have been discovered that not only produce increased melanin yields but also save energy and time and protect the thermolabile compounds in the extract [84].

The cavitation-based extraction technique is currently used as it is eco-friendly and does not require the use of hazardous solvents. Cavitation is a phenomenon in which cells develop a small, low-pressure vapor-filled cavity because of a sudden shift in pressure under a liquid medium. As cavitation bubbles collapse, an energy is generated that increases the mass transfer within the surfaces and improves the penetration of a solvent into the cellular materials [85].

These new methods include cavitation-based extraction techniques such as ultrasound-assisted extraction (UAE), negative pressure cavitation (NPC) extraction, microwave-assisted extraction (MWE), and hydrodynamic cavitation extraction (HCE).

### 6.1. Ultrasound-Assisted Extraction (UAE)

This technique utilizes ultrasound pressure waves to increase the efficacy of melanin extraction. In this process, a cavitation-induced mass transfer is generated in the fungal cell wall. As the cavitation bubbles are formed and collapsed, the generated energy induces an increase in the penetration of the solvent into the cell wall, due to which the cell wall is easily disrupted and the cellular matrix may be separated out [86].

The method has many advantages, such as increasing the extraction yield, reducing solvent usage, economizing power consumption, and shortening the duration of the extraction. The disadvantage of this technique is that the ultrasonic waves are concentrated on the material, and the dispersed extracted materials are not homogenous [87].

### 6.2. Negative Pressure Cavitation (NPC)

In the case of NPC, the creation of negative pressure governs cavitation. This extraction proved to be more effective in the extraction of heat-sensitive compounds like polyphenols and polysaccharides [88].

### 6.3. Microwave-Assisted Extraction (MWE)

Microwave-assisted extraction is one of the most practical methods utilized in industrial purposes due to the availability of equipment, its convenient handling, and its high extraction efficiency. This is another advanced technique that was discovered recently. In this method, microwave energy is generated that heats the solvent in contact with the fungal biomass and releases the bioactive compounds out of the fungal cell [88]. This method was first used in the extraction of melanin from *Lachnum singeranum* YM296. The quantity of melanin produced was significantly increased in *Lachnum singerianum* YM296 with the aid of this technique and other optimized parameters [89].

The difficulty with this method is that the extraction process ends early because the solvent boils and eventually increases the temperature of the reaction mixture. As a result, the extraction yield is decreased because the targeted chemicals are not sufficiently dispersed from the sample into the solvent [90].

### 6.4. Hydrodynamic Cavitation Extraction (HCE)

Cavitation occurs due to pressure variations in the flowing liquid with respect to the change in the geometry of the constriction [88].

However, every extraction method has its own pros and cons, and therefore, no standard protocols for the extraction of melanin have been described until now. Hence, the extraction methods, fermentation conditions, and optimization parameters can be coordinated accordingly to obtain increased yields of melanin from different fungal sources [91].

## 7. The Characterization of Melanin

Even after the successful extraction of the pigment, the chemical characterization of melanin remains a challenge. The pigment is highly heterogenous in nature due to the lack of a genetic makeup responsible for the biosynthesis of melanin and sequential metabolic pathways. Its physicochemical properties, such as its high heterogenicity, hydrophobicity, insolubility in organic solvents, and resistance to chemical degradation, make it difficult to unscramble its structure and restrict the techniques capable of identifying and characterizing the pigment [92].

**Figure 2:**
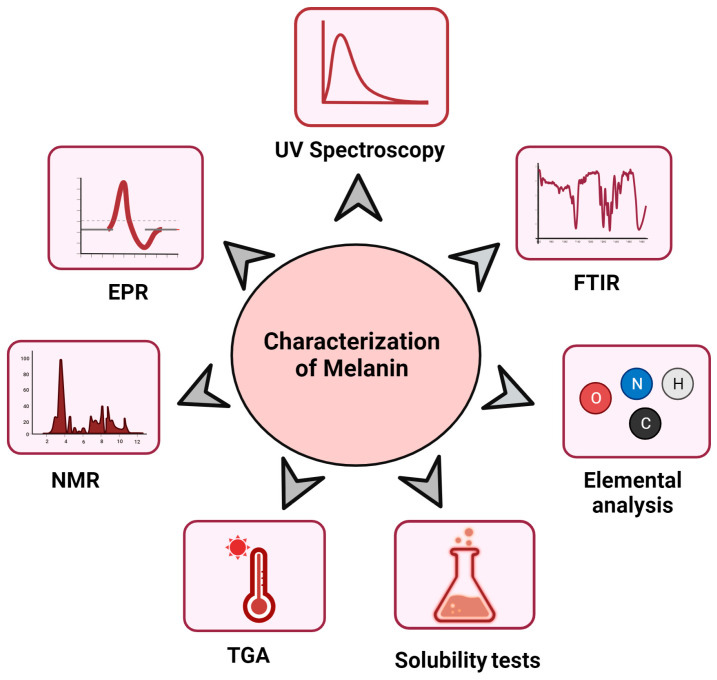
Various methods for the characterization of melanin.

With concerted efforts over time, it became possible to elucidate the proper structure of melanin using various techniques, such as UV and visible spectroscopy, elemental analyses, physico-chemical tests, FTIR (Fourier Transform Infra-Red Spectroscopy), EPR (Electron Paramagnetic Resonance), and NMR (Nuclear Magnetic Resonance) (Figure 2). At a primary stage, basic determinations of solubility and UV spectra can also provide relevant information in a short time. Due to the heterogenous nature of melanin, it lacks a well-defined structure. Therefore, meticulous characterization techniques are taken into account so as to ascertain the structure of melanin [93].

### 7.1. UV–Visible Spectroscopy

One of the characteristic methods for the identification of melanin in its preliminary stage is its maximum absorption in the UV-visible spectra (i.e., 200–400 nm). The complex conjugated molecules in the structure of melanin absorb and scatter UV light photons; for this reason, alkaline melanin solutions show significant optical absorption in the UV region (200–400 nm) which gradually declines toward longer wavelengths (400–800). This subsequent decline in absorption is almost linear. As a result, straight lines with negative slopes are formed when the logarithm of the absorbance of an alkaline melanin solution is plotted versus the wavelength. This linear connection forms a distinguishing characteristic for the qualitative identification of melanin in an extract [94].

### 7.2. Fourier Transform Infra-Red (FTIR) Spectroscopy

FTIR spectroscopy is another method for the detection of melanin in which functional groups are known. Through studying the structure of melanin, it is has already been established that eumelanins comprise polymeric units of indole-5,6-quinone and 5,6-dihydroxy-indole-2-carboxylic acid, and pheomelanins have benzothiazine and benzothiazole units in their structures. Therefore, the different functional groups in the heterocyclic rings of eumelanins and pheomelanins produce characteristic peaks in the spectra for the detection of the type of melanin [95].

There are some common characteristic peaks that differentiate eumelanins and pheomelanins. The typical bands found in the FTIR spectra of the range of melanin are in wavelength ranges from 3600 to 3000 cm^−1^, 1650 to 1600 cm^−1^, and 1500 to 1400 cm^−1^. The stretching vibrations of the -OH and -NH groups belonging to the amine, amide, carboxylic acid, phenolic, and aromatic amino functionalities present in the indolic and pyrrolic systems have been attributed to the strong, broad absorption band detected at 3600–3000 cm^−1^ [96]. The vibrations of the aromatic C=C and C=O stretches of carboxylic function are typically linked to the strong, distinctive absorption band between 1650 and 1600 cm^−1^. Between 1500 and 1400 cm^−1^, certain melanin stretches were observed: the bending vibration of N-H and the stretching vibration of C-N (a secondary amine) [97].

### 7.3. Elemental Analysis

Elemental analysis studies of melanin help us understand the type of melanin on the basis of the composition of elements and the elemental ratio. Eumelanins contain high C, H, and N contents and lack sulfur because they are synthesized via L-dopa or L-tyrosine oxidation. On the other hand, pheomelanins have high sulfur contents (9–12%) as they are products of cysteinyl conjugates of DOPA. Hence, the presence of sulfur in pheomelanins forms the characteristic difference between the two types of melanins [98].

### 7.4. Physicochemical Properties

Conventional physicochemical tests are typically used for the initial detection and characterization of melanin due to its distinctive solubility and reactivity characteristics. Melanin has a number of distinguishing characteristics, including a low level of solubility in distilled water, low levels of solubility in most organic and inorganic solvents (except for aqueous alkali), a resistance to degradation by concentrated acids, bleaching upon exposure to potassium permanganate, potassium dichromate, sodium hypochlorite, hydrogen peroxide, or other oxidizing agents, a positive reaction for polyphenols (via the FeCl_3_ test), reacting with sodium dithionite and potassium solutions, and reacting with ammoniacal silver nitrate solutions [99].

However, it is found to be soluble in DMSO, alkaline water (pH 10.0), and phosphate-buffered saline (pH 7.2). The solubility of melanin in alkali solvents is suitable for its extraction purposes since melanin is precipitated under acidic conditions [100].

### 7.5. Thermal Characterization

In comparison to other natural polymers, fungal melanin has greater heat stability. According to Gomez-Marin and Sanchez (2010), the graphite-like structure of the polymer is responsible for its thermal stability. Therefore, a thermogravimetric analysis (TGA) can be used to examine the heat stability of melanin. To assess the thermal behaviors of fungal melanins, TGA thermograms curves were utilized. Typically, a characteristic pattern of peaks is visible in the two stages of the heat deterioration of fungal melanin. First, an endothermal peak is formed due to the evaporation of bound water at approximately 250 °C. Following the endothermal peak, the second is an exothermal peak caused by the loss of CO_2_ that occurs at 350 °C [101]. At temperatures above 500 °C, melanin decomposition has been documented. Pheomelanins display two exothermic peaks in derivative thermogravimetric analyses (DTA) at temperatures of 200–300 °C and 500–560 °C, respectively. This is the primary distinguishing trait of pheomelanins In general, synthetic melanins are more thermally stable at high temperatures than natural melanins [102].

### 7.6. Nuclear Magnetic Resonance (NMR)

In order to further confirm the molecular structure of extracted melanin, a proton and carbon nuclear magnetic resonance (^1^H-NMR, ^13^C-NMR) analysis can be carried out. The sample is subjected to an NMR analysis using various resonant frequencies and is usually referenced to tetramethylsilane. There are several chemical shifts in the ^1^H-NMR spectra of melanin which can support the correct identification of the melanin’s molecular structure [103].

### 7.7. Electron Paramagnetic Resonance (EPR)

The EPR signal can be used to determine the existence of free radical centers in the structure of melanin, which are features of melanin biopolymers. EPR tests performed on pigmented soil fungi such as *Cladosporium cladosporioides* and mushroom fungi *Schizophyllum commune* have demonstrated the existence of melanin [104].

O-semiquinone radicals are produced by eumelanins, whereas o-semi quinone imine radicals are produced by pheomelanins. Even at higher microwave powers, the EPR spectrum of eumelanin displays a single line with a hyperfine form, whereas the EPR spectrum of pheomelanin exhibits a complicated shape with an unresolved structure, primarily due to the interactions between free radical electrons and the nearby nitrogen nuclei [105].

Another approach to analyzing the polymeric structure of melanin is pyrolysis gas chromatography coupled with mass spectrometry (py-GC-MS) as this technique degrades macromolecules by heating them to high temperatures, causing the dissociation of bonds [106].

In recent years, Raman spectroscopy has also proven to be an effective tool for understanding the structure of melanin. Strycker et al. [107] characterized Raman spectra of *Aspergillus fumigatus* (DHN melanin) and *Cryptococcus neoformans* (DOPA melanin), and it was interpreted that the spectra provided a “map” of the biosynthetic pathways for DHN- and DOPA-melanins.

Therefore, the proper characterization of melanin becomes important so that the pigment can be explored and utilized in a wide range of biotechnological applications in various fields of medicine, food processing, dermocosmetics, and bio-electronics (Table 1)**.**

## 8. Applications of Melanins

### 8.1. Applications in Bio-Electronic Industries

**Figure 3:**
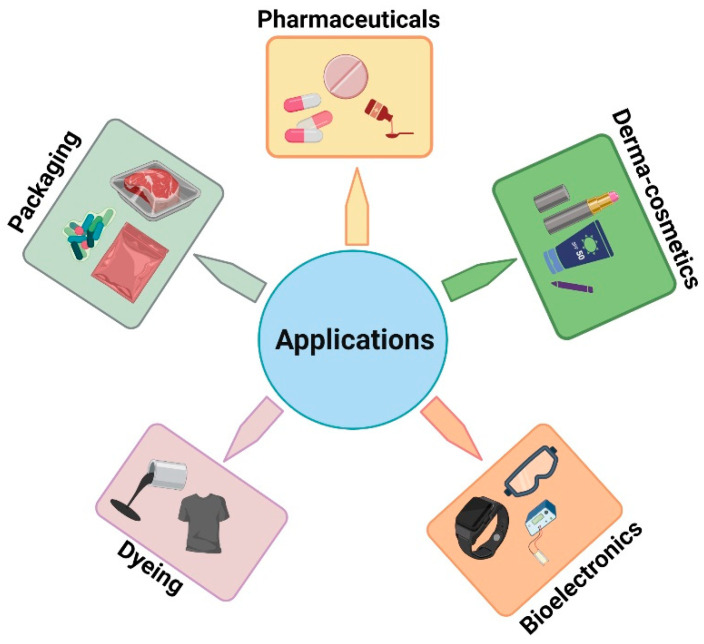
Applications of fungal melanin in various industries.

Many interesting optoelectronic properties of melanin have been studied, such as its high optical absorption in the UV-Vis range, good electronic transmission, and ionic conductivity, which makes it a potential biomaterial for use in organic electronic devices with a less negative impact on the environment. Studies suggest that the electrical conductivity properties of melanin highly resemble those of amorphous semiconductors; therefore, melanins are considered organic semiconductors [115]. These are cheaper and easier to process relative to inorganic semiconductors like silicon and germanium and could therefore be used as a promising materials in sensors and photovoltaic devices (Figure 3) [116].

Owing to the optical and electronic properties of melanin, thin films have been produced from these pigments that can be used in solar cells, chemo-sensors, and many other detectors. Melanins are also employed as sensitizing pigments in DSSCs (dye-sensitized solar cells), which transform photons into their electrically excited state [117]. The ultraviolet absorption property of melanin pigment has also been extremely utilized in the manufacture of ophthalmic devices, protective eyewear packaging material, umbrellas, and other materials that protect from harmful radiation. Moreover, melanin has also been employed as a composite material in the aerospace industry to shield spacecraft from the risks of radiation exposure [118]. It can also be used to safeguard astronauts and radiation therapy patients. Studies also suggest that the melanin found in black edible mushrooms can be utilized as a patient-friendly oral radioprotectant to reduce the harmful effects of radiation on human health [119].

### 8.2. Applications in the Dyeing Industries

The biodegradable nature of fungal melanin as an eco-friendly dye demonstrates its potential uses in natural hair colors and cosmetics. On a similar note, the durability of fungal melanin for dyeing purposes was demonstrated via the melanin released by *Lasiodiplodia theobromae*, which was used to tint bleached poplar veneers. The photoprotective and antioxidant potentials of fungal melanin are also attributed to its application in the textile industry [120], e.g., a recent study conducted by Ahn et al. [121] showed that allomelanin from the actinomycete *S. glaucescens* was used to dye cotton fabrics. An experiment suggested that a laccase enzyme treatment significantly improved the intensity of the dye.

### 8.3. Applications in Pharmaceutical Industries

Due to its promising antioxidant and anti-inflammatory effects, fungal melanin has been explored and widely utilized in drug development, therefore offering a fresh approach to boosting antioxidant therapy [122]. It has been reported to treat various types of malignant tumors in cancer, disorders of the immune system, diseases of blood origin, disorders due to disturbances in cell homeostasis, mental disorders such as schizophrenia, epilepsy, and other disorders involving the nervous and other regulatory systems [113,114]. The effective free radical scavenging ability of melanin enhances immunological function, triggers apoptosis, and prevents blood angiogenesis; for these reasons, fungal melanin has been developed as a powerful anticancer therapeutic component [79]. Owing to its ability to increase the permeability of the blood–brain barrier, melanin is also useful as a carrier for other therapeutic agents which must reach the brain tissue to produce their therapeutic responses. Melanin nanoparticles have been explored as potential biocompatible drug carriers, especially in relationd to pH targeting as it responds strongly to pH [123]. Experiments on melanin extracted from the fungus *Schizophyllum commune* resulted in the successful inhibition of the proliferation of the HEP-2 cell line [109]. It has been suggested that fungal melanin could possibly be used against cancer and as a tool in chemotherapy. Hou et al. [111] studied the strong genoprotective effects of melanin from *Inonotus hispidus* and suggested its potential to be applied for the development of anti-carcinogenic preparations.

### 8.4. Applications in the Dermocosmetic Industry

In the dermocosmetic industry, melanin has especially been a subject of keen interest as it was found to have great potential when incorporated into skin photoprotection formulations, especially sunscreens that protect against the noxious effects of UV radiation [124]. The emerging demand for safer and chemically inert dermocosmetic products has encouraged the use of melanin as an active ingredient in sunscreens. This property of melanin is attributed to the high efficiency of the pigment in absorbing and scattering photons, simultaneously quenching the radicals in their excited state and scavenging reactive oxygen species [125].

In two separate studies using two different melanins, Kurian and Bhat compared the SPF values of commercially available sunscreens with the effect of melanin on the enhancement of the SPF value, demonstrating that melanin increased the SPF value by factors of 3.42 and 2.6 [126,127]. An allomelanin extracted from the black knot fungus also proved to be a great choice for cosmetics and as an anti-UV radiator [108]. Similarly, the saprophytic fungi *Lachnum* produces large amounts of melanins via submerged fermentation. They have also been observed to serve as natural anti-radiation additives in sunscreens as they indicated increased chances of survival against the bacteria *E. coli* and *S. aureus* and the yeast *Saccharomyces cerevisiae* under UV radiation [112].

In recent years, efforts have been taken to produce melanin that is soluble in an aqueous cosmetic buffer so as to make the use of this pigment as an ingredient in cosmetic lotions, creams, ointments, etc. Though melanin-based cosmetic products are currently available on the market, this market is yet to be explored, and the demand for natural, melanin-incorporating products is likely to increase.

### 8.5. Applications in Packaging Materials

The packaging industry has also seen an increase in the manufacturing of composite chemical materials made from fungal melanin. This potential of fungal melanin has been attributed to its antibacterial and antioxidant properties. Thin films developed from carvacrol and fungal melanin are effectively sold in the market, providing a future alternative to plastic films for green packaging and food packaging [128].

Fungal melanin can also function as a coating agent for material surface modifications since it adheres well to the surfaces of many different materials, such as metals, polymeric materials, ceramics, biological surfaces, and mineral complexes, to generate functional materials. This explains its potential to evolve into a useful packaging material [129]. Some of the most important aspects of food processing and packaging technologies include good thermal stability and a heat-processing ability. The melanin synthesized from *Chroogomphus rutilus* showed effective stability at a temperature of 80–100 °C for 5 h, in sunlight for 40 days, and under UV light for 240 min, demonstrating the potential applications of this fungus in the food industry [110].

## 9. Future Approaches

### 9.1. The Use of Statistical Methods

Traditional methods of optimization that use one variable at a time are tedious, dull, and produce inconsistent yields because they ignore the interface between the elements of production that are being investigated. Therefore, the limitations of traditional approaches need to be overcome via newly developed statistical experimental design tools such as PBD and RSM for the optimization of the conditions of melanin biosynthesis [130].

In the statistical modeling approach, important factors are selected from a large number of concurrently examined factors. The most frequently applied method for identifying the significant factors is the Plackett–Burman design (PBD) in which significant factors are chosen for optimization tests while non-significant factors (such as a type of melanin that shows a very low effect on response values) are excluded from experiments. On the other hand, the response surface methodology (RSM) approach is used to examine the relationship between the tested variable and the response (melanin production) after screening the design and associated outcomes. In order to accommodate a range of experimental settings, including the quantity and concentration of the investigated components, the characteristics of the design space, and the number of trials employed, RSM includes a number of fixable designs. The two most popular methods for the optimization and maximization process are the Central Composite Design (CCD) and Box–Behnken Design methods [131], e.g., the increased production of melanin in *Auricularia auricula* was investigated using the PBD and CCD methods. Under the established ideal conditions, the melanin yield was 1.08 g/L in comparison to 306.52 mg/L at suboptimal conditions, showing a 3.52-fold increase [132]. Therefore, this statistical technique creates an efficient fermentation process that speeds up the biosynthesis of melanin at a reduced cost.

### 9.2. The Use of Economical Substrates

Major efforts are involved in making the production of melanin more cost-effective by employing agro-industrial waste as an eco-friendly substitute. The literature should focus more on developing simple methods that produce melanin semi-industrially while avoiding the use of costly chemicals. Utilizing agro-industrial waste as a substrate for fermentation is another strategy that is still uncommon among research studies. The residues from agro-industrial biomass are easily accessible and economically viable as fermentation substrates for microbial melanin biosynthesis [109].

### 9.3. The Production of Water-Soluble Melanin

A major restriction on the application of melanin is due to its insoluble nature. However, water-soluble melanin is more effective in a variety of biotechnological applications. For this reason, the solubilization of melanin using a variety of techniques has been the focus of study in many scenarios to increase the utilization of melanin.

Ghadge et al. [133] studied the use of tetrabutylammonium hydroxide (40% *w*/*w* TBAOH in water) to extract melanin from the actinomycete *Streptomyces hyderabadensis* 7VPT5-5R. The aqueous TBAOH method increased the yield of melanin by 66% compared to conventional methods. Similarly, the solubilization of melanin using ultrasonic pressure increased the propagation of ultrasound pressure waves and the ensuing cavitation phenomena, suggesting that the solubility of melanin may not be connected to its structural deterioration [134]. As a result, certain additional methods for the ultrasonic degradation process were employed to speed up the decomposition of a polymer while maintaining its undamaged state. These methods included using ultrasound in conjunction with an enzyme or using ultrasound in an alkaline and hydrogen peroxide environment [135].

### 9.4. The Copper-Mediated Expression of Tyrosinase-Encoding Genes

Copper is important for the biosynthesis of both DHN- and DOPA-melanin as it serves as a cofactor for the enzymes tyrosinases and laccases. It can also be said that copper affects the pathways of melanin biosynthesis by regulating the expression of these enzymes [136].

In addition, physical factors like pH, temperature, incubation period, and media components also regulate gene expression and therefore affect biosynthesis. As a result, these circumstances are typically changed according to the specific melanogenic strains. The cryptic biosynthetic pathways of melanin can be activated and deactivated by altering the conditions, thereby also altering the expression of the genes. As a result, fermentation conditions and genetic alteration are closely connected, and both are important factors in improving melanin biosynthesis [137].

### 9.5. The Use of Melanin Nanoparticles

Melanin nanoparticles (NPs) have also been shown to have promising improved capabilities over their natural form in the biomedical area. The use of NPs in the development of novel medicines is proving to be a practical choice in the biomedical industry. NPs have higher levels of selectivity and specificity for a given target than microparticles because they have larger surface areas and can modify their surfaces. Melanin nanocarriers may be used in medicine not only as a diagnostic tool in photothermal therapy but also in chemotherapy with controlled drug release; this dual action is referred to as theranostics [138].

After entering the circulatory system, these melanin NPs can enter cells via receptor-mediated transcytosis or endocytosis; hence, these NPs are also capable of improving drug-loading capacity and stimulating regulated drug delivery [139].

Additionally, melanin NPs are strongly biocompatible, easier to eject from common organs like the liver and kidneys, and have fewer harmful effects when they accumulate over time in organs [140].

## 10. Conclusions

Due to its multifarious properties, melanin has been widely recognized for its potential in diverse applications. Among micro-organisms, fungi have emerged as a great source of this ubiquitous pigment as they are easier to upscale on the industrial level while maintaining diversity. However, knowing the potential applications of fungal melanins, they are still executed successfully very often. There lies a complexity in the structure of these biopolymers which is mainly due to the enzymatic imbalances in the biosynthetic pathways that alter their metabolic routes. This heterogenous nature of melanin is responsible for making the extraction and chemical characterization of the pigment difficult. With concerted efforts, a massive scope of work has been completed to circumvent challenges in the isolation of melanin and techniques for elucidating its structure. The development of new extraction methods that do not involve harsh treatments has enabled efficient extraction, and many analytical techniques have also been explored to successfully determine the structure of melanin. The optimization of the media used in fermentation also plays an important role in increasing the bioproduction of melanin. This comprehensive review covers all the aspects of melanins produced by fungi. Though fungal melanin has a promising future in the dermatological, biomedical, cosmetic, agricultural, and environmental technology fields, it has yet to reach to its full potential. Research in fungal melanin has a bright future, specifically to improve the bioproduction of melanin to achieve industrial-level yields and to utilize the pigment in biotechnology to a great extent for societal benefits.

## Figures and Tables

**Table 1:** List of fungi that produce melanin.

Fungi That Produce Melanin	Salient Findings Regarding Melanin	Reference
*Amorphotheca resinae*	The study examined melanin production from *A. resinae*, achieving 4.5 g/L in 14 days, with potent antioxidant properties and structural characterization via elemental analysis and spectroscopy.	[103]
*Armillaria mellea*	Isolated and characterized melanin from *A. mellea* rhizomorphs displayed antioxidant, light barrier, and antibacterial properties.	[56]
*Aspergillus bridgeri*	The study identified a melanin pigment from *A. bridgeri*, confirming its identity via FTIR and EPR spectroscopy, with potential applications in the cosmetics and pharmaceutical industries.	[94]
*Auricularia auricula*	The study used a low-cost fermentation medium with wheat bran extract, L-tyrosine, and CuSO_4_ for melanin production in *A. auricula*.	[46]
*Auricularia auricula*	The study examined melanin production in *A. auricula*, finding the highest growth rates in low-carbon and carbon-free media and low yields in nitrogen-free media.	[86]
*Apiosporina morbosa*	The study revealed melanin extracted from *A. morbosa*, a pathogenic black knot fungus, with a 10% yield. This nitrogen-free allomelanin is low-cost and invasive, making it an alternative green source for UV light absorbers and antioxidants.	[108]
*Aspergillus fumigatus*	*A. fumigatus*, an immunosuppressed fungal pathogen, produces DHN melanin and alternative pyomelanin through a different pathway, confirming the identity as pyomelanin through the deletion of essential enzymes.	[109]
*Aspergillus nidulans*	The study identified a melanin-type pigment extracted from *A. nidulans*, revealing physical and chemical properties similar to synthetic DOPA-melanin. Tricyclazole and tropolone inhibit melanin production.	[100]
*Chroogomphus rutilus*	*C. rutilus* produces melanin with UV absorption, FTIR, and chemical reactions, offering potential applications in the food, pharmaceutical, and cosmetic industries.	[110]
*Cryomyces antarcticus*	The study found the potential of melanin from *C. antarcticus* in radioprotection research, offering potential applications in bioremediation and biomedical fields.	[62]
*Exidia nigricans*	The study investigated melanin from *E. nigricans*, focusing on its isolation, characterization, and color properties. Purified melanin showed better light properties and higher antioxidant activity.	[57]
*Gliocephalotrichum simplex*	UV, ^13^C, and ^1^H NMR spectra characterized an extracellular melanin pigment from *G. simplex*; tyrosine and peptone supplementation enhanced melanin production up to 6.6 g/L.	[48]
*Humicolopsis cephalosporioides*	The study investigated the environmental factors affecting chlamydospore differentiation and pigment biosynthesis in *H. cephalosporoides*, finding that temperature and light influence the development and melanization essential for survival in sub-Antarctica soils.	[106]
*Inonotus hispidus*	The study characterized *I. hispidus* melanin using solid-state fermentation and ultrasonic-assisted extraction, revealing its antioxidant activity.	[111]
*Inonotus obliquus*	*I. obliquus* studies showed increases melanin complex production under submerged conditions, with potential antioxidant and genoprotective effects.	[47]
*Lachnum* YM404	LEM404-A extracellular melanin exhibits a strong UV radiation activity, increasing bacterial survival rates against *Escherichia coli*, *Staphylococcus aureus,* and *Saccharomyces cerevisiae.*	[112]
*Lachnum singerianum*	The microwave-assisted extraction of melanin from *L. singerianum* YM296 increased its yield by 11.08% and 40.43% compared to alkali and acid precipitation. LIM-a showed anti-aging activity in aged mice, enhancing body weight and reducing MDA levels.	[64]
*Phoma* sp. RDSE17	*Phoma* sp. RDSE17 melanin exhibits antioxidant, anti-microbial, and anticancer properties, with low nitrogen content and high DPPH-free radical-scavenging activity.	[53]
*Phyllosticta capitalensis*	*P. capitalensis* produces DHN-melanin, a pigment crucial for its survival in stressful environments, which was characterized via UV, IR, and ESR tests.	[67]
*Pleurotus cystidiosus*	The study identified melanin in edible *P. cystidiosus* mushrooms and black coremea produced by *Antromycopsis macrocarpa.*	[113]
*Phomopsis*	The pigment extracted from the endophyte *Phomopsis* was characterized to be a DOPA type of melanin.	[114]
*Schizophyllum commune*	Extracellular melanin from an *S. commune* mushroom fungus showed significant antibacterial, antifungal, and concentration-dependent HEP-2 inhibition.	[51]
*Scleroderma citrinum*	The study investigated the biological properties of raw and purified melanins from *S. citrinum*, finding that purified melanins have better light properties and antioxidant activity.	[58]
*Spissiomyces endophytica*	The study examined melanin production and characterization from *S. endophytica* using UV, FTIR, EPR, and chemical tests, revealing a low nitrogen content.	[49]

## Data Availability

The data that support this study are available from the corresponding author upon reasonable request.
